# Paratesticular Liposarcoma: A Case Report and Review of the Literature

**DOI:** 10.1155/2013/806289

**Published:** 2013-02-28

**Authors:** Haider Alyousef, Elsawi M. Osman, Mohamed A. Gomha

**Affiliations:** Urology Department, King Fahad Specialist Hospital, Dammam 15215, Saudi Arabia

## Abstract

*Introduction*. Liposarcoma is a rare pathological entity. By far it is the most common histological subtype of genitourinary sarcomas in adults. Approximately two hundred cases were reported in the literature. We are hereby presenting a case with a typical clinical scenario of paratesticular liposarcoma. *Case report*. A 75-year-old gentleman presented with a painless right hemiscrotal swelling that was progressively increasing in size over the last 6 years. Testicular tumour markers were negative. Imaging showed a heterogenous mass with fat component. Subsequently he underwent wide local excision that included the paratesticular mass along with the right testicle and all right inguinal canal contents up to the deep inguinal ring with the sparing of right illioinguinal nerve. Histopathological examination showed a well differentiated liposarcoma of the spermatic cord. He remained recurrence-free so far after 18 months of followup. *Conclusion*. Radical orchidectomy with wide local excision comprises the cornerstone of treatment of paratesticular liposarcoma. Due to the rarity of the disease there is no definite universal consensus of opinion as regards the role of radiotherapy and chemotherapy.

## 1. Case Report 

A 75-year-old gentleman presented to our patient clinic with a history of right scrotal swelling for almost 6 years. It was painless and slowly increasing in size with no relation to the patient's posture. He also gave a history of mixed voiding and storage lower urinary tract symptoms. Physical examination revealed a nontender mass which was firm anteriorly with softer posterior aspect. The right testicle was felt distinct from the mass with normal size and consistency. The mass was neither reducible nor transilluminating. The left testis was also normal. 

**Figure 1:**
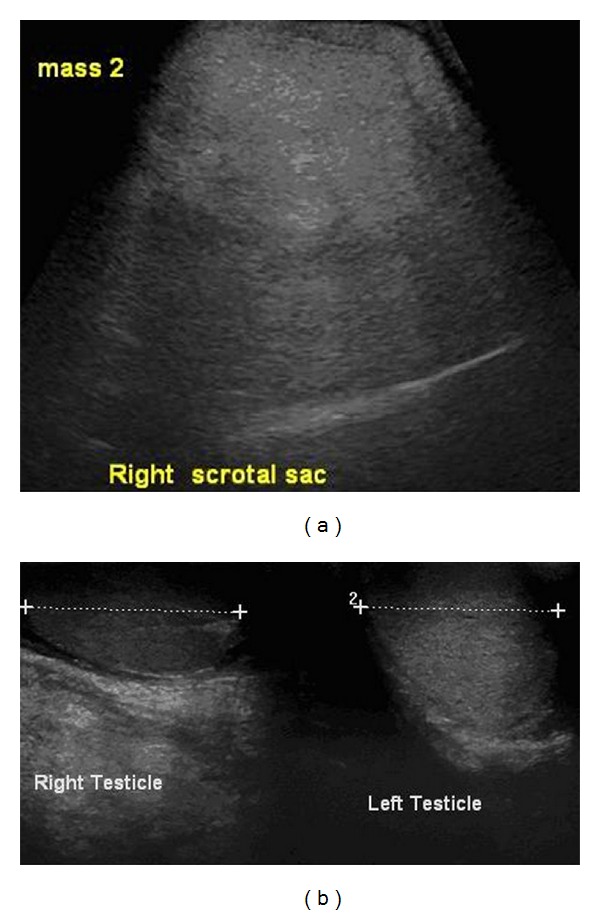
Bilateral normal testes (a) with a hetrogenous mass in the right hemi-scrotum (b).

Laboratory investigations including testicular tumor markers were normal. Scrotal ultrasound showed a large right scrotal heterogeneous mass, mainly hyperechoic in echotexture and measuring 8.5 × 5.4 cm. Both testicles were sonographically normal (Figure 1). 

**Figure 2:**
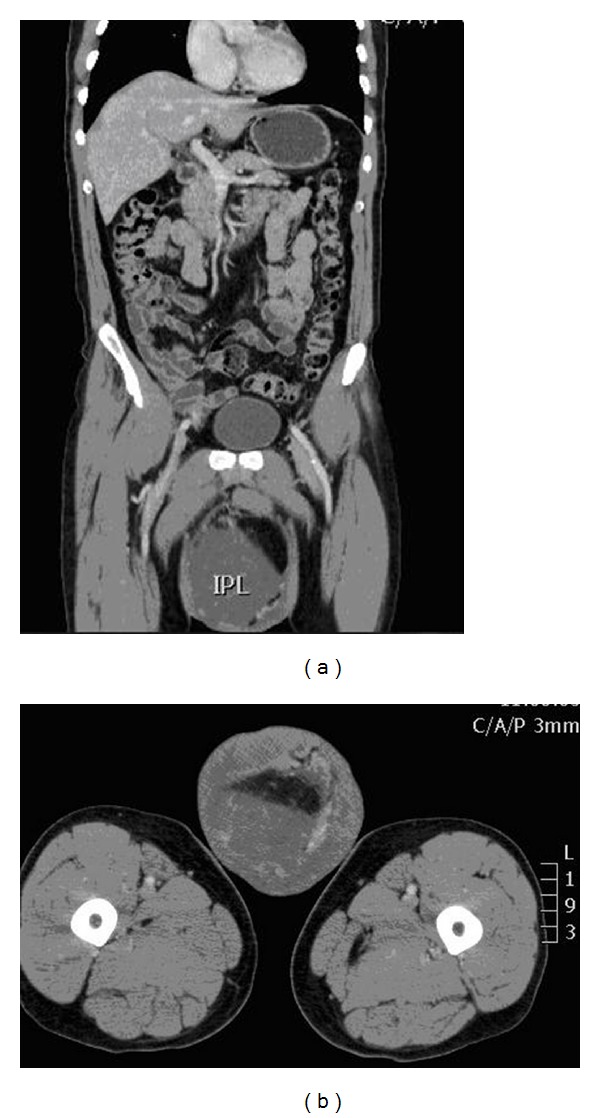
Coronal and axial views of CT abdomen and pelvis (a and b) showing the heterogenous mass with fat component.

CT scan with IV contrast revealed a right-sided scrotal sac multifocal fat-containing lesion extending deeply to right inguinal canal. No lymphadenopathy and no distant metastasis were detected (Figure 2).

**Figure 3:**
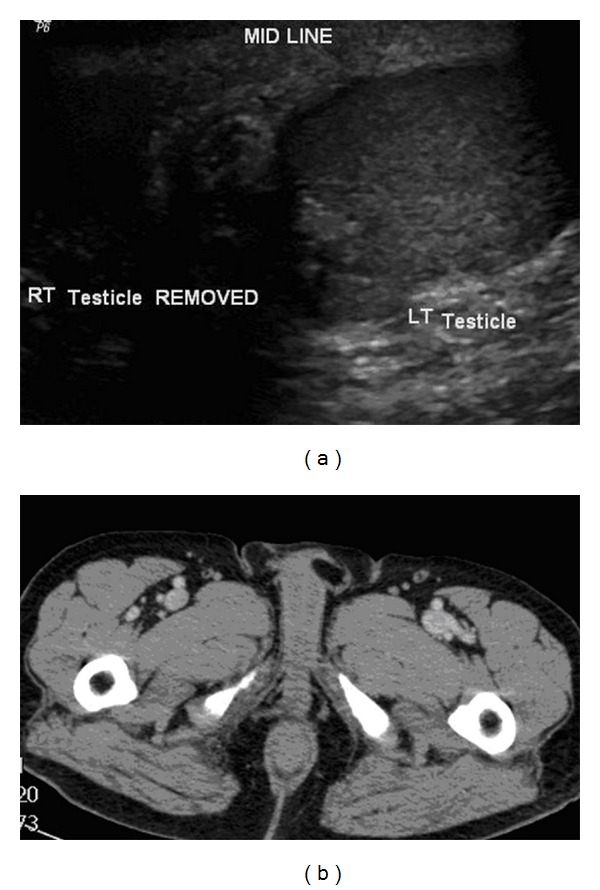
Follow up US and CT scans (a and b) showing no evidence of local recurrence.

Consequently, a wide local excision that included the paratesticular mass along with the right testicle and all right inguinal canal contents up to the deep inguinal ring with the sparing of right illio-inguinal nerve was carried out through an inguinal incision. Histopathological examination revealed a well-differentiated liposarcoma of the spermatic cord with a normal testicle. The patient made a satisfactory postoperative recovery. On two subsequent clinic visits, there was no evidence of local recurrence clinically. Follow-up scrotal ultrasound and CT scan of the abdomen and pelvis with IV contrast (Figure 3) revealed no evidence of local recurrence, lymphadenopathy, or distant metastasis. Further follow-up plan will comprise of 6 monthly clinic visits for clinical examination with surveillance CT scan performed annually. Resection of any amenable recurrences with adjuvant radiation therapy will be our preferred modality of treatment should the disease recur.

## 2. Discussion 

Para-testicular liposarcoma is a rare entity making it difficult to have a universal consensus on the natural history and management even in large institutions. [1, 2] Less than 200 cases were reported up to date. 

Spermatic cord is the most common site followed by testicular tunics and epididymis (76%, 20%, and 4%, resp.). Average age at presentation is 55 years (range 16.5 to 85). The above clinical scenario represents the typical mode of presentation. Occasionally, para-testicular liposarcoma presents as a rapid increase in the size of previously stable mass. In less than 6% of cases there is history of scrotal surgery or trauma. The presentation can be mistaken for inguinal hernias or hydrocele. [3] Few cases have been reported to be associated with retroperitoneal involvement 

Para-testicular liposarcoma appears as a nonhomogeneous mass with variable echoes consistent with fat. CT scan with IV contrast is an excellent radiological tool for diagnosis. It is used preoperatively and postoperatively for staging and followup, respectively. 

Based on histological appearance liposarcomas in general have been classified into myxoid (most common; 40%), round cell, well differentiated (subdivided into lipoma-like, sclerosing, inflammatory and dedifferentiated), and pleomorphic. [4–6] Liposarcomas tend to spread primarily by local extension. Hematogenous and lymphatic spread is usually a late event exhibited by high-grade tumors. 

With regards to treatment radical orchiectomy and en bloc removal of the tumor with wide local excision are the standard treatment. It has been reported that no therapeutic advantage could be attributed to superficial inguinal or retroperitoneal lymphadenectomy. 

Radiation therapy has been used for local control in liposarcoma in general. Liposarcomas are the most radiosensitive sarcoma and in some cases remission has been achieved with radiotherapy alone but the results in para-testicular liposarcoma are less clear. In some studies radiotherapy in 11 cases has been associated with recurrence in 5 cases. Recurrence after radiotherapy may have a higher-grade and more aggressive neoplasm with dedifferentiation. Radiation therapy is recommended in addition to surgery [5] in situations with evidence of tumor with propensity for more aggressive behavior (i.e., if inadequate margin, recurrence, highgrade or if there is lymphatic invasion). 

There is a paucity of data regarding the results of chemotherapy in metastatic para-testicular liposarcoma but the results of chemotherapy for liposarcoma at other sites are poor. Adjuvant chemotherapy has been reported in 2 cases but followup has been short. Doxorubicin chemotherapy has been used occasionally. 

Para-testicular liposarcoma has a high propensity for local recurrence, the commonest reason for which is incomplete resection. The reported recurrence rate ranges between 46% and 57%. Prognosis and survival vary in relation to histopathological classification. Myxoid and well-differentiated have better prognosis than round cell and pleomorphic liposarcoma. Five-year survival rates are 80% and 20%, respectively. 

## 3. Conclusion 

 Even in large institutions, it is difficult to accumulate sufficient cases to document the natural history for such a tumor and draw conclusions about treatment results because of rarity of the tumor. When diagnosed or suspected preoperatively, radical orchiectomy with wide local excision is the recommended treatment. Adequate local resection provides the best chance of eradicating this disease. Adjuvant radiation should be considered in tumors of intermediate or high grade and in recurrent liposarcoma after previous wide excision. The role of chemotherapy is not well defined. Regardless of initial therapy, the risk of local recurrence and subsequent increase in grade always necessitates long-term followup.
